# Fast quantum control in dissipative systems using dissipationless solutions

**DOI:** 10.1038/s41598-019-39731-z

**Published:** 2019-03-11

**Authors:** François Impens, David Guéry-Odelin

**Affiliations:** 1 Instituto de Física, Universidade Federal do Rio de Janeiro, Rio de Janeiro, RJ 21941-972 Brazil; 2 Université de Toulouse, UPS, Laboratoire Collisions Agrégats Réactivité, IRSAMC, F-31062 Toulouse, France; 3 CNRS, UMR 5589, F-31062 Toulouse, France

## Abstract

We report on a systematic geometric procedure, built up on solutions designed in the absence of dissipation, to mitigate the effects of dissipation in the control of open quantum systems. Our method addresses a standard class of open quantum systems that encompasses non-Hermitian Hamiltonians. It provides the analytical expression of the extra magnetic field to be superimposed to the driving field in order to compensate the geometric distortion induced by dissipation for spin systems, and produces an exact geometric optimization of fast population transfer. Interestingly, it also preserves the robustness properties of protocols originally optimized against noise. Its extension to two interacting spins restores a fidelity close to unity for the fast generation of Bell state in the presence of dissipation.

## Introduction

The dynamical control and the preparation of well-defined quantum states with a high degree of accuracy and fidelity is a prerequisite for several important applications. In Nuclear Magnetic Resonance (NMR)^1,2^ or in Nitrogen-Vacancy(NV) center^3^ experiments, the accurate control of quantum spins is essential. The generation of entangled states is of special interest for their use as resources in various contexts such as quantum computing^4^, quantum cryptography^5^ or quantum metrology^6^. For instance, extremely accurate optical clocks using the entanglement between ions^7^ have been realized^8,9^.

In spite of these achievements, engineering entangled states with massive particles is still a challenging experimental task. Indeed, undesirable interactions of the quantum system with its environment unavoidably take place during the preparation stage, which tend to spoil the fidelity of the final state with respect to the target quantum state. The effects of such parasitic couplings increase with time, so that their influence may be attenuated by accelerating the quantum state preparation. For this purpose, shortcut to adiabaticity (STA) protocols^10^ have been used successfully in various contexts^11–18^. STA protocols have been proposed for the generation of entangled states with atomic spins^19–24^. Unfortunately, this acceleration comes at the price of a significant energy overhead. A perfect fidelity obtained through an extremely short time of preparation would generally require an unrealistic amount of energy.

Here, we combine STA protocols with a fine-tuning of the control parameters mitigating the effects of dissipation during the quantum state preparation to reach high fidelities with realistic parameters. We setup a systematic procedure to adapt in open quantum systems protocols optimized for dissipationless systems. It consists in maintaining the original geometry of an optimal quantum path in a dissipative environment by a proper engineering of the control fields.

We first discuss one-body quantum systems. For spin 1/2-like quantum systems, we show that a magnetic field correction, involving a moderate overhead of resources, enables one to compensate exactly the effects of the dissipation onto the average spin orientation. The correcting field only depends on the geometry of the trajectory and on the spin-field coupling constant, and not on the details of the magnetic or electric fields used to generate the trajectory. The preservation of the quantum trajectory on a Bloch sphere is exact and non-perturbative. An important benefit of our method concerns Stimulated Raman Adiabatic Passage (STIRAP)^25–27^. Among other applications, STIRAP has proven to be a key element for the formation of ultra-cold molecules^28,29^. We show below how our procedure may enable a fast and reliable STIRAP in the presence of dissipation. Interestingly, our procedure also preserves the robustness to noise in protocols originally designed in the absence of dissipation and involving the interaction of a two-level system with a noisy laser source.

This one-body procedure can be successfully transposed to more complex interacting quantum systems. Precisely, we show how the effects of dissipation in the quantum trajectories of two interacting spins controlled by a single magnetic field can be dramatically attenuated. We apply this approach to the fast generation of entangled Bell states in an environment presenting linear dissipation. For convenience, we shall model the effect of dissipation through non-Hermitian Hamiltonians contributions. Non-Hermitian Hamiltonians, eventually corrected through an effective procedure^30^, provide indeed a satisfactory simplified treatment of several phenomena such as superradiance^31^, transport^32^ in open quantum systems. Nevertheless, the method presented here could be adapted to account for the full quantum master equation.

## Results

### Cancellation of dissipation anisotropy through a fine-tuning of the driving field

We expose below our procedure, which relies on the adaptation of the driving field to a given dissipative environment. Starting from a control field inducing a quantum trajectory in a dissipationless environment, we build up a correction which depends on the geometry of the quantum trajectory and on the structure of the linear dissipation. Up to a renormalization factor, this technique enables one to preserve exactly the quantum trajectory in spite of the dissipation. This approach is non-perturbative and involves only a moderate overhead of energetic resources.

#### Problem statement

We consider the interaction of a spin 1/2 with a time-dependent magnetic field, following the Hamiltonian $\hat{H}(t)=-\,\gamma \,\hat{{\bf{s}}}\cdot {\bf{B}}(t)$ with the spin operator ${\bf{s}}=\hslash \hat{\sigma }\mathrm{/2}$ defined through the Pauli matrices *σ*_*k*_ for *k* = *x*, *y*, *z*. *γ* is the gyromagnetic factor. The average spin value ${\bf{S}}(t)=\langle \hat{{\bf{s}}}\rangle (t),$ follows a precession equation about the magnetic field. In several experimental situations discussed below, this precession equation must be complemented by a dissipation term:

1 $$\frac{d{\bf{S}}}{dt}=\gamma \,{\bf{B}}\times {\bf{S}}-\overline{\overline{{\rm{\Lambda }}}}\,{\bf{S}}$$

**Figure 1:**
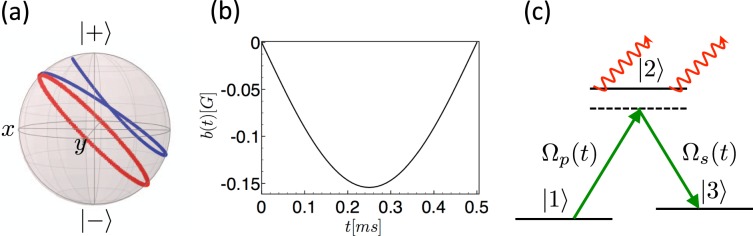
(**a**) Dissipationless (red curve) vs dissipative (blue curve) trajectory on the Bloch sphere of a spin-1/2 particle subjected to a 2*π*-pulse. Dissipation is modeled by the tensor $\overline{\overline{{\rm{\Lambda }}}}={{\rm{\Gamma }}}_{\perp }(\hat{{\bf{x}}}\hat{{\bf{x}}}+\hat{{\bf{y}}}\hat{{\bf{y}}})$. Since the magnetic field correction and the renormalized trajectory would be unaffected by an additional isotropic dissipation, this model indeed captures any linear dissipation tensor with a degenerate eigenvalue. The initial state density matrix is $\rho \mathrm{(0)}=\frac{1}{2}+\frac{1}{2\sqrt{2}}({\sigma }_{x}+{\sigma }_{z}),$ and we apply a constant magnetic field ${\bf{B}}=\frac{{B}_{0}}{\sqrt{2}}(\,-\,\hat{{\bf{x}}}+\hat{{\bf{z}}})$. We have renormalized the spin norm to unity for sake of clarity (${{\rm{\Gamma }}}_{\perp }=0.7\times (\gamma {B}_{0})$). (**b**) Magnetic field correction $b(t)={\bf{b}}(t)\cdot {\hat{{\bf{u}}}}_{\phi }(t)$ (in G) as a function of time (ms) for a *π*-pulse induced by a constant magnetic field. We have used Eq. (3) and considered the transverse relaxation time ${T}_{2}^{\ast }={{\rm{\Gamma }}}_{\perp }^{-1}\,\simeq \,2\,{\rm{ms}}$, taken ${{\rm{\Gamma }}}_{z}\ll {{\rm{\Gamma }}}_{\perp }$ and a pulse duration *T* = 0.5 ms, corresponding to typical parameters of an hyperpolarized Helium −3 NMR experiment^36^ (gyromagnetic ratio *γ* = 32.4 M*Hz*/*T*^59^). (**c**) STIRAP transfer between the levels |1〉 and |3〉 through an intermediate level |2〉 undergoing a dissipative process.

$\overline{\overline{{\rm{\Lambda }}}}$ is the second rank tensor with positive real eigenvalues accounting for the dissipation. This model captures well the interaction of a two-level quantum system with a generic Markovian environment with no restrictions on the dissipation rates (Different approaches may be used when considering the interaction with optical cavities^22–24^ or wave-guides^33^, or in the presence of a non-Markovian environments with a suitable noise spectrum^34^). The effect of dissipation on a spin-1/2 trajectory is illustrated on Fig. 1a. Consider a magnetic field profile **B**_0_(*t*) designed to induce a given continuous average spin trajectory **S**_0_(*t*) on the Bloch sphere in the absence of dissipation between the instants *t* = 0 and *t* = *T* (**S**_0_(*t*) is solution of Eq. (1) with $\overline{\overline{{\rm{\Lambda }}}}=0$). We now ask the question: can one adjust the magnetic field to maintain the average spin trajectory **S**_0_(*t*) when $\overline{\overline{{\rm{\Lambda }}}}\ne 0$? A magnetic field modification cannot compensate for the damping of the average spin caused by the dissipative term along the prescribed trajectory. Nevertheless, as explained below, a fine-tuning of the magnetic field may correct the change of spin orientation due to dissipation.

#### Principle of our method

We introduce a renormalized average spin $\tilde{{\bf{S}}}(t)={\bf{S}}(t)\exp [F(t)]$, and look for a renormalization function *F*(*t*) and a magnetic field correction **b**(*t*) = **B**(*t*) − **B**_0_(*t*) such that the renormalized spin $\tilde{{\bf{S}}}(t)$ follows the original trajectory **S**_0_(*t*). For this purpose, the free parameters *F*(*t*) and **b**(*t*) need to fulfill the relation (Supplementary Material).

2 $$\dot{F}(t){{\bf{S}}}_{0}(t)+\gamma \,{\bf{b}}(t)\times {{\bf{S}}}_{0}(t)=\overline{\overline{{\rm{\Lambda }}}}\,{{\bf{S}}}_{0}(t)$$

where the dot denotes a time derivative. For an isotropic tensor $\overline{\overline{{\rm{\Lambda }}}}={\rm{\Lambda }}\,\overline{\overline{1}}$, the solution of Eq. (2) reads **b**(*t*) = 0 and *F*(*t*) = Λ*t*.

The magnetic field correction, **b**(*t*), indeed only addresses the anisotropy of the dissipation. The renormalization rate $\dot{F}(t)$ is unique and determined by the projection of the right hand side of Eq. (2) onto the spin **S**_0_(*t*). In contrast, the solutions **b**(*t*) for the corrective magnetic field can be chosen among a straight line $\{{{\bf{b}}}_{0}(t)+\lambda {{\bf{S}}}_{0}(t)|\lambda \in {\mathbb{R}}\}.$ **b**_0_(*t*) is a particular solution chosen without loss of generality such that **b**_0_(*t*)⋅**S**_0_(*t*) = 0 at all times. This freedom in the choice of the correction **b**(*t*) is reminiscent of the infinity of possible driving fields in the transitionless quantum driving method proposed by Berry^35^. Equation (2), together with the choice *F*(0) = 0, guarantees that the initial spin **S**_0_(*t*) and the renormalized spin $\tilde{{\bf{S}}}(t)$ trajectories are solutions of the same differential equation with the same initial condition. These two solutions thus coincide at any time during the interaction with the magnetic field. Up to a decay of the spin norm, one can thus maintain the original spin trajectory in the presence of dissipation by a fine adjustment of the magnetic field. We stress that this result is exact and non-perturbative.

#### Explicit evaluation of the correction

To illustrate our method, we evaluate the magnetic field correction for a general trajectory **S**_0_(*t*) and with a dissipation tensor exhibiting different transverse Λ_*xx*_ = Λ_*yy*_ = Γ_⊥_ and longitudinal ${{\rm{\Lambda }}}_{zz}={{\rm{\Gamma }}}_{//}$ relaxation rates. Such anisotropy occurs in NMR^1,2^ and NV center^3^ experiments, where the quantum spin longitudinal relaxation time *T*_1_ is usually several orders of magnitude larger than the transverse relaxation time *T*_2_. To determine the magnetic field correction **b**_0_(*t*), we use a decomposition on the spherical coordinate basis $({\hat{{\bf{S}}}}_{0}(t),{\hat{{\bf{u}}}}_{\theta }(t),{\hat{{\bf{u}}}}_{\phi }(t))$ with the unit vector ${\hat{{\bf{S}}}}_{0}(t)=(\sin \,\theta (t)\cos \,\phi (t),\,\sin \,\theta (t)\sin \,\phi (t),\,\cos \,\theta (t))$ corresponding to the average spin direction. From Eq. (2), one obtains

3 $${\bf{b}}(t)=\frac{{{\rm{\Gamma }}}_{//}-{{\rm{\Gamma }}}_{\perp }}{2\gamma }\,\sin \,2\theta (t)\,{\hat{{\bf{u}}}}_{\phi }(t),$$

which provides a non zero correction only in the anisotopic case. This correction cancels when the spin points towards the poles or crosses the equatorial plane. At these specific times, the average spin is indeed an eigenvector of the dissipation tensor, which preserves the spin orientation. An example of such time-dependent magnetic field correction, with parameters taken from an hyperpolarized Helium −3 NMR experiment^36^, is depicted on Fig. 1.

#### Energy considerations

We now discuss the energy overhead induced by our magnetic field correction. For our method, the amplitude of the magnetic field correction scales as the maximal difference between the dissipation tensor eigenvalues, and is completely determined by the spin orientation. In particular, it is unaffected by the average spin damping and is also independent of the magnetic field strength used to generate the dissipationless trajectory. We consider a *π*-pulse in a system with negligible longitudinal dissipation ${{\rm{\Gamma }}}_{z}\ll {{\rm{\Gamma }}}_{\perp },$ as often observed in NMR spectroscopy^1,2^. Precisely, we require that the final spin orientation be exactly along the axis *Oz*, but partially relax the constraint on the spin norm by imposing only ||***S***(*T*)||/||***S***(0)|| ≥ 1 − *ε* for a fixed *ε* > 0 at the final time *T* (left undetermined a priori). We take as dissipationless spin trajectory an ordinary *π* pulse involving a constant magnetic field **B**_0_, and evaluate the minimum energy ${E}_{\pi {\rm{corr}}\mathrm{.}}=\frac{1}{2}{\int }_{0}^{T}dt||{\bf{B}}(t){||}^{2}$ associated to the corrected magnetic field **B**(*t*) = **B**_0_ + **b**(*t*). The damping of the average spin sets an upper bound on the total time *T*. The minimum energy takes the form of two additive contributions ${E}_{\pi }=-{\pi }^{2}{\gamma }^{-2}{{\rm{\Gamma }}}_{\perp }/[4\,\mathrm{ln}(1-\varepsilon )]$ and ${\rm{\Delta }}{E}_{\pi }=-\,\frac{1}{8}{\gamma }^{-2}{{\rm{\Gamma }}}_{\perp }\,\mathrm{ln}(1-\varepsilon ),$ respectively associated to the constant magnetic field and to the magnetic field correction (Supplementary Material). In the low-damping limit $\varepsilon \ll 1$, adequate description of most NMR experiments, the overhead induced by our magnetic correction becomes a small fraction of the total energy as ${\rm{\Delta }}{E}_{\pi }/{E}_{\pi {\rm{corr}}\mathrm{.}}\simeq {\varepsilon }^{2}/(2{\pi }^{2}).$

### Application to fast population transfer

The procedure described above is particularly well-suited to enhance the performance of fast population transfer in open quantum systems. We show indeed that our method drastically enhances the efficiency of a STIRAP population transfer and avoids the contamination of the final state by the initial or/and intermediate states despite the presence of dissipation. As discussed below, an additional key feature of our procedure is that it preserves the benefits of a previous optimization toward a noise source.

#### Fast Stimulated Raman Adiabatic Passage

STIRAP enables robust population transfers between two states, denoted |1〉 and |3〉, which are both coupled to a third intermediate state |2〉 with two quasi-resonant fields. Differently from the usual STIRAP protocols, in the quantum-accelerated STIRAP protocols^16,37–39^ the system quantum state may experience a significant overlap with the intermediate state |2〉 during the transfer. This distinctive feature turn these quantum-accelerated STIRAPs more fragile to a dissipation process involving the intermediate state. In a Λ-level configuration, the intermediate state has a higher energy and may thus suffer a stronger dissipation than the lower levels |1〉 and |3〉. As discussed below, our procedure provides a significant enhancement of these quantum protocols in such a dissipative three-level system.

We consider the Λ-level configuration of Fig. 1b where only the intermediate state |2〉 is subject to dissipation, corresponding to a transfer outside the multiplicity {|1〉,|2〉,|3〉}, and modelled by a non-Hermitian Hamiltonian ${\hat{H}}_{\Gamma }=-i\hslash {\rm{\Gamma }}\mathrm{|2}\rangle \langle \mathrm{2|}$. As our procedure is immune to an isotropic dissipation, the discussion below would also address a configuration with two equally dissipative levels |1〉,|3〉. Within the Rotating Wave Approximation (RWA) and in the interaction picture, the control Hamiltonian associated to the resonant field pulses reads ${\hat{H}}_{0}(t)=\frac{\hslash }{2}[{{\rm{\Omega }}}_{p}(t)\mathrm{|1}\rangle \langle \mathrm{2|}+{{\rm{\Omega }}}_{s}(t\mathrm{)|2}\rangle \langle \mathrm{3|]}+{\rm{h}}{\rm{.c}}.,$ with Ω_*p*_(*t*) and Ω_*s*_(*t*) the Rabi frequencies of the pump and Stokes fields respectively. The Schrödinger equation for the state |*ψ*(*t*)〉 = *C*_1_(*t*)|1〉 + *C*_2_(*t*)|2〉 + *C*_3_(*t*)|3〉 with ${\hat{H}}_{0}(t)$ boils down to a precession equation for a pseudo-spin ${\bf{S}}(t)=-{C}_{3}(t)\hat{{\bf{x}}}-i{C}_{2}(t)\hat{{\bf{y}}}+{C}_{1}(t)\hat{{\bf{z}}}$ involving a pseudo-magnetic field ${\bf{B}}(t)=\frac{1}{2}[{{\rm{\Omega }}}_{p}(t)\hat{{\bf{x}}}+{{\rm{\Omega }}}_{s}(t)\hat{{\bf{z}}}]$^40^. The Hamiltonian ${\hat{H}}_{{\rm{\Gamma }}}$ results in an additional dissipation tensor $\overline{\overline{{\rm{\Lambda }}}}={\rm{\Gamma }}\,\hat{{\bf{y}}}\hat{{\bf{y}}},$ turning the precession equation into Eq. (1). In the fast STIRAP protocol, the system quantum state follows an eigenstate |*ψ*_0_(*t*)〉 of a dynamical Lewis-Riesenfeld invariant parametrized as $|{\psi }_{0}(t)\rangle =\,\cos \,\gamma (t)\cos \,\beta (t\mathrm{)|1}\rangle -i\,\sin \,\gamma (t)\mathrm{|2}\rangle -\,\cos \,\gamma (t)\sin \,\beta (t)\mathrm{|3}\rangle .$ The correction to the pseudo-magnetic field ${\bf{b}}(t)=\frac{1}{2}[\delta {{\rm{\Omega }}}_{p}(t)\hat{{\bf{x}}}+\delta {{\rm{\Omega }}}_{s}(t)\hat{{\bf{z}}}]$, following the procedure above, corresponds to a change in the Rabi frequencies $\delta {{\rm{\Omega }}}_{p}(t)=-{\rm{\Gamma }}\,\sin \,2\gamma (t)\cos \,\beta (t)$ and $\delta {{\rm{\Omega }}}_{s}(t)={\rm{\Gamma }}\,\sin \,2\gamma (t)\sin \,\beta (t)$. Using the simple dissipationless fast STIRAP based on the second quantum protocol of ref.^37^ with the parameters *ε* = 0.05 and *δ* = *π*/4 in a dissipative system such that Γ*T* = 1.0, one obtains a final state with a fraction of roughly 6.5% in the states |1〉 and |2〉 (see Supplementary Material). Using the dissipationless fast STIRAP corrected by our procedure, one obtains only the desired final state with strictly no overlap with the initial and intermediate states.

#### Preservation of the robustness to noise

Several methods have been developed to optimize the control of two-level systems against noise, either due to the thermal environment^41^ or to the field driving the system^42^. We investigate here the effect of our procedure on a quantum protocol of fast population transfer in a two-level atomic system originally optimized against the amplitude noise of a laser source and in the absence of any dissipation. As discussed below, our procedure preserves the benefits of the optimization towards this noise source, while improving the population transfer in the presence of an additional dissipation process.

Following ref.^42^, the dynamics of a two-level atomic system controlled by the noisy laser field are adequately described by a Bloch equation of the form (1) involving an effective magnetic field $${\bf{B}}(t)={{\rm{\Omega }}}_{R}(t)\hat{{\bf{x}}}+{{\rm{\Omega }}}_{I}(t)\hat{{\bf{y}}}+$$$${\rm{\Delta }}(t)\hat{{\bf{z}}}$$ and a dissipation tensor accounting for the laser amplitude noise $${\overline{\overline{{\rm{\Lambda }}}}}_{{\rm{Laser}}}(t)=\frac{1}{2}{\lambda }^{2}[{{\rm{\Omega }}}_{I}^{2}(t)\hat{{\bf{x}}}\hat{{\bf{x}}}\,+\,$$$${{\rm{\Omega }}}_{R}^{2}(t)\hat{{\bf{y}}}\hat{{\bf{y}}}+$$$$({{\rm{\Omega }}}_{I}^{2}(t)+{{\rm{\Omega }}}_{R}^{2}(t))\hat{{\bf{z}}}\hat{{\bf{z}}}]$$. Ruschhaupt *et al*.^42^ have obtained optimally robust STA for the population inversion, that maximize the robustness against laser amplitude noise within a large set of fast quantum transfer protocols. We take as initial Bloch vector trajectory **S**_0_(*t*) an optimal shortcut described in spherical coordinates by $$\theta (t)=$$$$\pi t/T-\frac{1}{12}\,\sin (2\pi t/T),\phi (t)=\pi \mathrm{/4},$$ and implemented by resonant laser pulses (Δ(*t*) = 0) of time-dependent Rabi frequencies $${{\rm{\Omega }}}_{R}^{(\mathrm{opt})}(t)={{\rm{\Omega }}}_{I}^{(\mathrm{opt})}(t)=-\dot{\theta }(t)/\sqrt{2}.$$

**Figure 2:**
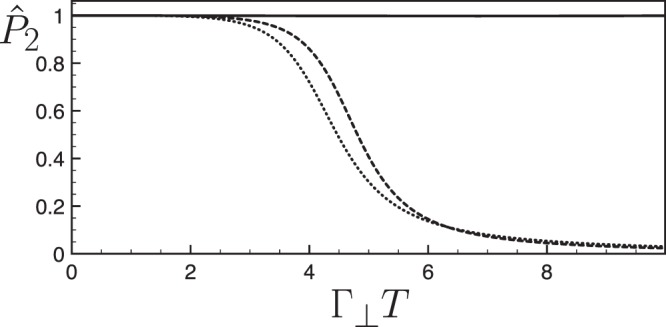
Values of the normalized probability ${\hat{P}}_{2}({{\rm{\Gamma }}}_{\perp }T)$ transfer probabilities as a function of the transverse attenutaion Γ_⊥_*T* for different protocols: standard *π*-pulse (dotted line), optimal shortcut toward the laser amplitude noise (dashed line), and optimal shortcut modified by our procedure (solid line). We have taken the strength of the laser noise as *λ* = 0.3.

We consider a situation where, in addition to the laser noise, the Bloch vector experiences a constant transverse dissipation tensor $\overline{\overline{{\rm{\Lambda }}}}={{\rm{\Gamma }}}_{\perp }(\hat{{\bf{x}}}\hat{{\bf{x}}}+\hat{{\bf{y}}}\hat{{\bf{y}}})$. We compare the efficiency of the optimal protocol modified by our procedure to both the uncorrected protocol and to a simple *π*-pulse. The transfer efficiency is estimated using the normalized probability ${\hat{P}}_{2}=\frac{1}{2}(1-{S}_{z}(T)/||{\bf{S}}(T)||)$ in the excited state at the final time *T*. By construction this quantity is insensitive to an isotropic damping and equal to unity for a perfect transfer. Figure 2 reveals that the dissipationless optimal protocol is improved by our procedure for a broad range of transverse dissipations. In the strongly dissipative regime, the transverse damping induces a final Bloch vector almost parallel or antiparallel to the *Oz* axis. Above a critical value of the transverse dissipation, the flip of the Bloch vector is inhibited for the uncorrected protocols, while it is preserved thanks to our procedure. In the presence of a transverse attenuation Γ_⊥_*T* = 6 and a laser amplitude noise corresponding to *λ* = 0.3, one obtains the respective transfer probabilities ${p}_{2}^{(\pi )}=0.455$, ${p}_{2}^{(\mathrm{opt}.)}=0.465$ and ${p}_{2}^{(\mathrm{opt}\mathrm{.}/{\rm{c}})}=0.532$ for a standard *π*-pulse, for the optimal shortcut and for the optimal shortcut improved by our procedure. Beyond the specific protocol considered here, our method can be implemented to mitigate the effects of dissipation in different families of STA trajectories, optimized toward strong noise sources^43^ or toward the presence of unwanted transitions^44^.

### Application to fast generation of entangled states

We now discuss the benefits of our procedure for the fast generation of two-qubits entangled states in open quantum systems. Quantum teleportation, relying on the ability to prepare entangled states, has early been considered as an important resource for scalable quantum architectures^45^. More generally, single and two-qubits quantum operations are the building blocks for quantum information processing in experimental architectures involving spin chains and arrays^46–53^. Any enhancement of the quantum fidelity in these basic operations is thus important for larger quantum information processing tasks.

We consider a system of two identical spins-$\frac{1}{2}$ controlled by a single magnetic field and interacting through an Ising potential ${\hat{V}}_{{\rm{int}}}^{(dd)}=4\xi \,{\hat{S}}_{1z}{\hat{S}}_{2z}$ with the operator ${\hat{S}}_{mz}$ accounting for the *z*-component of the spin *m* with eigenvalues $\pm \hslash \mathrm{/2}$. The Hamiltonian, $\hat{H}=-\gamma ({\hat{{\bf{S}}}}_{1}+{\hat{{\bf{S}}}}_{2})\cdot {\bf{B}}(t)+{\hat{V}}_{{\rm{int}}}^{(dd)}$, is invariant under the permutation of labels 1 and 2. As a result, the symmetric subspace $\{|\,++\rangle ,|{\rm{Bell}}\rangle =\frac{1}{\sqrt{2}}(|+-\rangle +|-+\rangle ),|--\rangle \}$ is stable during the evolution. The adiabatic passage technique can be used to generate an entangled Bell state from a fully polarized state^54^, and involves a careful design of the time-dependent magnetic field in order to decouple the subspace $\{|++\rangle ,|\mathrm{Bell}\rangle \}$ from the state $|\,--\rangle$. The magnetic field is engineered to avoid energy crossings, which would otherwise jeopardize the adiabaticity conditions ensuring the stability of this subsystem. The stability of the subsystem $\{|\,++\rangle ,|{\rm{Bell}}\rangle \}$ is indeed a mere approximation valid only for sufficiently slow variations of the magnetic field, differently from the stability of the subspace $\{|\,++\rangle ,|{\rm{Bell}}\rangle ,|\,--\rangle \}$ which is exact. With this technique, a Bell state can be reliably generated from a fully polarized state in a typical time of ${T}_{{\rm{adiabatic}}}\gtrsim 30\hslash /\xi$ for a magnetic field strength $B\simeq 0.8\xi /(\hslash \gamma )$. The use of STA^19^ provides a speed-up of roughly one order of magnitude^20,21^. For shorter generation times, the two-dimensional subspace $\{|\,++\rangle ,|{\rm{Bell}}\rangle \}$ is no longer stable and thus the fidelity decreases. This shortcut is implemented using the superposition of a rotating transverse magnetic field ${{\bf{B}}}_{\perp }(t)=B(t){\rm{Re}}[(\hat{{\bf{x}}}+i\hat{{\bf{y}}}){e}^{i\omega t}]$ and a time-dependent longitudinal field ${{\bf{B}}}_{//}(t)={B}_{z}(t)\hat{{\bf{z}}}$ obtained from a reverse engineering method within the subspace $\{|\,++\rangle ,|{\rm{Bell}}\rangle \}$^19,20^ (Supplementary material).

In the following, we assume that the fully polarized and the Bell spin states suffer dissipative processes with different relaxation rates $${{\rm{\Gamma }}}_{|++\rangle }$$ and Γ_|Bell〉_, described by the non-Hermitian Hamiltonian $${\hat{H}}_{{\rm{\Gamma }}}=-i\hslash {{\rm{\Gamma }}}_{|++\rangle }|\,++\rangle \langle ++|$$$$-i\hslash {{\rm{\Gamma }}}_{|{\rm{Bell}}\rangle }|{\rm{Bell}}\rangle \langle {\rm{Bell}}|$$. The ratio between these two relaxation rates, considered here as fixed parameters, may depend on coherence effects such as superradiance. In order to design the magnetic field correction for the shortcut trajectory, we focus on the quantum motion within the $$\{|\,++\rangle ,|{\rm{Bell}}\rangle \}$$ subspace, considering only the associated reduced density matrix. The generation of a Bell state from the fully polarized state corresponds to a simple population inversion within this subspace. The equation of motion for the reduced density matrix involves a commutator of the density matrix for the Hermitian part of the Hamiltonian and an anti-commutator for the non-Hermitian part. Stricly speaking, the quantum motion occurs within a space isomorphic to $${{\mathbb{R}}}^{4}$$. This is so because the trace of the reduced density matrix in the $$\{|\,++\rangle ,|{\rm{Bell}}\rangle \}$$ multiplicity is damped by dissipative processes. Nevertheless, in a perturbative treatment of the dissipation, one may treat to leading order this trace as an invariant of motion. The quantum motion can then be captured by the usual three-dimensional Bloch vector picture by using a projection of the reduced density matrix on the three Pauli matrices. This yields an equation of motion for the Bloch vector involving a precession due to the magnetic field and an additional constant drift induced by the dissipation anisotropy. Finally, we find the corresponding magnetic field correction by considering the undamped Bloch vector motion (Supplementary material). A similar perturbative approach still holds for the Optical Bloch Equations while they cannot be accounted for by a non-Hermitian Hamiltonian^55^. The associated Bloch vector follows indeed a precession equation involving simultaneously a linear anisotropic dissipation tensor and a constant drift.

**Figure 3:**
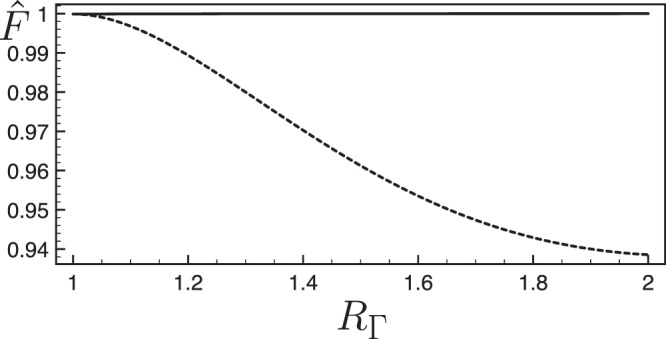
Renormalized fidelity $\hat{F}=|\langle {\rm{Bell}}|\psi (\tilde{T})\rangle {|}^{2}/|\langle \psi (\tilde{T})|\psi (\tilde{T})\rangle {|}^{2}$ obtained in the generation of the entangled state as a function of the ratio ${R}_{\Gamma }={{\rm{\Gamma }}}_{|++\rangle }/{{\rm{\Gamma }}}_{|{\rm{Bell}}\rangle }$ with (solid line) or without (dashed line) correction. We have taken $T=100\hslash /\xi$ and ${{\rm{\Gamma }}}_{|{\rm{Bell}}\rangle }T=2.5$.

Beyond the dissipation, non-adiabatic couplings between the subspace $\{|\,++\rangle ,|{\rm{Bell}}\rangle \}$ and the state $|\,--\rangle$ may also spoil the fidelity of the Bell state generation. We consider scenario for which the additional quantum state $|\,-\,-\rangle$ is undamped. We investigate the efficiency of our method by performing numerical simulations of the Schrödinger equation in the full subspace $\{|\,++\rangle ,|{\rm{Bell}}\rangle ,|\,--\rangle \}$ accessible from the the initial state $|\,+\,+\,\rangle$, and study the renormalized Bell state fidelity $\hat{F}=|\langle {\rm{Bell}}|\psi (T)\rangle {|}^{2}/|\langle \psi (T)|\psi (T)\rangle {|}^{2}$ as a function of the dissipation anisotropy characterized by the ratio ${R}_{{\rm{\Gamma }}}={{\rm{\Gamma }}}_{|++\rangle }/{{\rm{\Gamma }}}_{|{\rm{Bell}}\rangle }$ between the relaxation rates. As the anisotropy increases, the renormalized fidelity decreases in the uncorrected quantum protocol whereas it remains close to unity with our trajectory correction procedure (see Fig. 3). The quantum protocol improved by our method achieves a pure Bell state by filtering out efficiently the $\{|\,++\rangle ,|\,--\rangle \}$ states.

## Discussion

Our analytical approach builds up quantum protocols in dissipative systems from their dissipationless counterpart. It is based on the preservation of the geometric motion of a quantum state vector on the Bloch sphere and addresses dissipative processes described by non-Hermitian Hamiltonians. The resource overhead required to implement the corrected control fields is small. It successfully enhances the efficiency of fast STIRAP transfers in a dissipative environment. Interestingly, our modified protocol can preserve an optimization made in a dissipationless context. Our approach could thus be particularly relevant in situations where shortcuts to adiabaticity are used jointly with optimal control protocols^56–58^. This procedure has been extended to quantum systems involving two interacting quantum spins. Our method for enhancing the quality of two-spin entanglement could be fruitfully incorporated within single or two-qubit operations in larger quantum architectures^46–53^.

## Supplementary information

Supplementary Material (pdf)
