# Supplementary material for: Fast quantum control in dissipative systems using dissipationless solutions
Source: Sci Rep. 2019 Mar 11;9:4048. doi: 10.1038/s41598-019-39731-z (PMC6412050; doi:10.1038/s41598-019-39731-z)
Supplement: Supplementary file 1 — Supplementary Material (pdf) [file 41598_2019_39731_MOESM1_ESM.pdf]

Suppplementary Information for

Fast quantum control in dissipative systems using dissipationless solutions

François Impens[1,+], David Guéry-Odelin[2,3,*]

[1]Instituto de Física, Universidade Federal do Rio de Janeiro, Rio de Janeiro, RJ 21941-972, Brazil
[2] Université de Toulouse, UPS, Laboratoire Collisions Agrégats Réactivité, IRSAMC, F-31062 Toulouse, France
[3] CNRS, UMR 5589, F-31062 Toulouse, France

Corresponding authors: [+]impens@if.ufrj.br, [*]dgo@irsamc.ups-tlse.fr

This PDF file inlcludes:

- Additional details on the derivation performed in the article.
- An additional Figure showing the efficiency of the correction of dissipation in the quantum-enhanced STIRAP.

Cancellation of dissipation anisotropy through a fine-tuning of the driving field

Principle of our method

For sake of clarity, we recall here the equations of the main text related to our approach. We consider an average spin $\mathbf{S}_0(t)$ following the precession equation

$$\frac{d\mathbf{S}_0}{dt} = \gamma \mathbf{B}_0 \times \mathbf{S}_0 \tag{1}$$

We seek to adjust the magnetic field in order to obtain the same average trajectory, up to a renormalization factor, for the motion of an average spin $\mathbf{S}(t)$ in the presence of a linear dissipation term. The corresponding equation of motion takes the form

$$\frac{d\mathbf{S}}{dt} = \gamma \mathbf{B} \times \mathbf{S} - \overline{\overline{\Lambda}}\mathbf{S} \tag{2}$$

where we have noted $\mathbf{B}(t) = \mathbf{B}_0(t) + \mathbf{b}(t)$ the total magnetic field including a correction $\mathbf{b}(t)$ to be determined. One considers the renormalized average spin $\tilde{\mathbf{S}}(t) = \mathbf{S}(t)\exp[F(t)]$. By construction and by virtue of Eq.(2), the renormalized spin $\tilde{\mathbf{S}}(t)$ follows the equation of motion

$$\frac{d\tilde{\mathbf{S}}}{dt} = \gamma \mathbf{B}_0 \times \tilde{\mathbf{S}} + \dot{F}\,\tilde{\mathbf{S}} + \gamma \mathbf{b} \times \tilde{\mathbf{S}} - \overline{\overline{\Lambda}}\tilde{\mathbf{S}} \tag{3}$$

One chooses $F(t)$ and a magnetic field $\mathbf{b}(t)$ such that, at all time $t > 0$:

$$\dot{F}(t)\mathbf{S}_0(t) + \gamma \mathbf{b}(t) \times \mathbf{S}_0(t) = \overline{\overline{\Lambda}}\mathbf{S}_0(t) \tag{4}$$

The existence of a real function $\dot{F}(t)$ and a vectorial function $\mathbf{b}(t)$ ensuring this condition follows from elementary linear algebra considerations. Condition (4) determines the function $F(t)$ up to a constant, and one may set

$$F(t) = \int_0^t dt'\,\mathbf{S}_0(t')\cdot\overline{\overline{\Lambda}}\mathbf{S}_0(t')\,. \tag{5}$$

in order to obtain $\tilde{\mathbf{S}}(0) = \mathbf{S}_0(0)$. Thanks to the precession equation (2) and to the condition (4), the trajectory $\mathbf{S}_0(t)$ is also a solution of Eq. (3). The functions $\mathbf{S}_0(t)$ and $\tilde{\mathbf{S}}(t)$ are indeed solutions of the same differential equation with the same initial condition. They thus coincide at any time, so that $\mathbf{S}(t) = \mathbf{S}_0(t)\exp[-F(t)]$ for $t \geq 0$.

The magnetic correction $\mathbf{b}(t)$ can be obtained from Eq. (4). It is convenient to introduce the spherical basis $(\hat{\mathbf{S}}_0(t), \hat{\mathbf{u}}_\theta(t), \hat{\mathbf{u}}_\varphi(t))$ and use the angular parametrization

$$\hat{\mathbf{S}}_0(t) = \sin\theta(t)\cos\varphi(t)\mathbf{x} + \sin\theta(t)\sin\varphi(t)\mathbf{y} + \cos\theta(t)\mathbf{z}\,. \tag{6}$$

In the specific case where the dissipation tensor has a degenerate eigenvalue,

$$\overline{\overline{\Lambda}} = \Gamma_\perp(\hat{\mathbf{x}}\hat{\mathbf{x}} + \hat{\mathbf{y}}\hat{\mathbf{y}}) + \Gamma_z\hat{\mathbf{z}}\hat{\mathbf{z}} \tag{7}$$

the magnetic field correction yields

$$\mathbf{b}(t) = \frac{\Gamma_z - \Gamma_\perp}{2\gamma}\,\sin 2\theta(t)\,\hat{\mathbf{u}}_\varphi(t) \tag{8}$$

This example captures in particular many relevant experimental situations where dissipation is mostly transverse.

Energy considerations

We obtain here the energy overhead associated to the magnetic field correction for a simple π pulse. Following the discussion of the main text, we seek to realize a spin inversion such that at the final time T

$$||\mathbf{S}(T)||/||\mathbf{S}(0)|| \geq 1 - \varepsilon \tag{9}$$

for a given $\varepsilon > 0$ in a system presenting a purely transverse linear dissipation $\overline{\overline{\Lambda}}$ of the form (7) with $\Gamma_z = 0$. The total time T is *a priori* a free parameter.

Without loss of generality, we consider a trajectory $\hat{\mathbf{S}}_0(t)$ parametrized by $\theta(t) = \pi t/T$ e $\varphi(t) = 0$. In a dissipationless system, this trajectory can be induced by a constant magnetic field $\mathbf{B}_0 = \pi(\gamma T)^{-1}\,\hat{\mathbf{y}}$. The average spin orientation $\hat{\mathbf{S}}_0(t)$ can be

maintained in the dissipative system thanks to a total magnetic field $\mathbf{B}(t) = \mathbf{B}_0 + \mathbf{b}(t)$ involving the correction $\mathbf{b}(t)$ determined by our method in Eq. (8).

The damping of the spin norm is unaffected by the magnetic field correction. It is captured by the renormalization function (5). The considered trajectory $\mathbf{S}_0(t)$ and the transverse dissipation tensor $\overline{\overline{\Lambda}}$ yield $F(T) = \Gamma_\perp T/2$. The constraint (9) may thus be rewritten as an upper bound for the duration of the spin inversion:

$$T \leq -2\Gamma_\perp^{-1} \ln(1-\varepsilon) \tag{10}$$

The energy $E = \frac{1}{2}\int_0^T dt||\mathbf{B}(t)||^2$ associated to the total magnetic field reads $E = E_\pi + \Delta E_\pi$ where $E_\pi = \frac{1}{2}B_0^2 T = \pi^2/(2\gamma^2 T)$ and $\Delta E_\pi = \frac{1}{2}\int_0^T dt||\mathbf{b}(t)||^2 = \Gamma_\perp^2 T/(16\gamma^2)$ are the respective contributions of the constant magnetic field and of the magnetic field correction. The time minimizing the total energy $T_{\text{opt}} = \sqrt{8}\pi\Gamma_\perp^{-1}$ is always larger than the lower bound (10), except for extremely inaccurate spin inversion $\varepsilon \gtrsim 0.99$ of little physical interest. The minimal energy of a corrected π-pulse is thus obtained by saturating the bound (10), yielding the contributions $E_\pi = -\pi^2\gamma^{-2}\Gamma_\perp/[4\ln(1-\varepsilon)]$ and $\Delta E_\pi = -\frac{1}{8}\gamma^{-2}\Gamma_\perp \ln(1-\varepsilon)$ mentioned in the article.

Application to fast population transfer

Fast Stimulated Raman Adiabatic Passage

We provide here some additional details on the implementation of our method for the fast STIRAP protocol introduced by Chen and Muga in the absence of dissipation[1].

The system quantum state $|\psi(t)\rangle = C_1(t)|1\rangle + C_2(t)|2\rangle + C_3(t)|3\rangle$ follows a Schrödinger equation involving a control Hamiltonian $\hat{H}_0(t) = \frac{\hbar}{2}[\Omega_p(t)|1\rangle\langle 2| + \Omega_s(t)|2\rangle\langle 3|] + \text{h.c.}$ accounting for the interaction with the laser fields . In contrast with Ref.[1], we also take into account a non-Hermitian Hamiltonian $\hat{H}_\Gamma = -i\hbar\Gamma|2\rangle\langle 2|$ to model the dissipation suffered by the intermediate state $|2\rangle$. The corresponding equation boils down to a precession equation (2) for an effective spin $\mathbf{S}(t) = -C_3(t)\hat{\mathbf{x}} - iC_2(t)\hat{\mathbf{y}} + C_1(t)\hat{\mathbf{z}}$ interacting with an effective magnetic field

$$\mathbf{B}(t) = \frac{1}{2}[\Omega_p(t)\hat{\mathbf{x}} + \Omega_s(t)\hat{\mathbf{z}}] \tag{11}$$

and subject to a dissipation tensor $\overline{\overline{\Lambda}} = \Gamma\hat{\mathbf{y}}\hat{\mathbf{y}}$.

In the reverse engineering of Ref.[1], the system quantum state is maintained in a given eigenstate $|\psi_0(t)\rangle$ of a dynamical Lewis-Riesenfeld invariant. This eigenstate, parametrized as $|\psi_0(t)\rangle = \cos\gamma(t)\cos\beta(t)|1\rangle - i\sin\gamma(t)|2\rangle - \cos\gamma(t)\sin\beta(t)|3\rangle$, follows a well-defined trajectory. This yields a prescribed trajectory for the associated effective spin $\mathbf{S}_0(t) = \cos\gamma(t)\sin\beta(t)\hat{\mathbf{x}} - \sin\gamma(t)\hat{\mathbf{y}} + \cos\gamma(t)\cos\beta(t)\hat{\mathbf{z}}$ in the dissipationless system.

We have chosen the trajectory $\mathbf{S}_0(t)$ that corresponds to the second quantum protocol of Ref.[1]. The angular functions $\beta(t)$ and $\gamma(t)$ must satisfy a set of boundary conditions at the initial and final times in order to fulfill the requirements of the Lewis-Riesenfeld invariant method. Other boundary conditions are specific of this protocol and related to the cancellation of the pump and Stokes laser fields at the initial and final times. A last condition on the angle $\gamma(t)$ at the middle time $T/2$ determines the maximum population of the intermediate state $|2\rangle$. The boundary conditions are

$$\begin{aligned} &\gamma(0) = \varepsilon,\ \dot{\gamma}(0) = 0,\ \gamma(T) = \varepsilon,\ \dot{\gamma}(T) = 0 \\ &\beta(0) = 0,\ \beta(T) = \pi/2 \\ &\dot{\beta}(0) = 0,\ \dot{\beta}(T) = 0,\ \gamma(T/2) = \delta \end{aligned} \tag{12}$$

In this fast STIRAP protocol, the maximum population of the intermediate state $|2\rangle$ during the process corresponds to $p_2 = |\langle 2|\psi(T/2)\rangle|^2 = \sin^2\delta$. We choose a value of $\delta = \pi/4$ yielding $p_2 = 1/2$. This fast STIRAP protocol differs in this respect from the common and slow STIRAP, in which the intermediate state is not significantly populated. We determine the angular functions $\beta(t), \gamma(t)$ as the least-order polynomials in time satisfying the conditions above.

We now apply our procedure to restore the spin trajectory $\mathbf{S}_0(t)$ despite the dissipative process acting on the intermediate state. For this purpose, it is convenient to introduce the instantaneous orthonormal basis $(\mathbf{S}_0(t), \mathbf{v}_1(t), \mathbf{v}_2(t))$ with the vectors $\mathbf{v}_1(t)$ and $\mathbf{v}_2(t)$ defined as $\mathbf{v}_1(t) = \sin\gamma(t)\sin\beta(t)\hat{\mathbf{x}} + \cos\gamma(t)\hat{\mathbf{y}} + \sin\gamma(t)\cos\beta(t)\hat{\mathbf{z}}$ and $\mathbf{v}_2(t) = -\cos\beta(t)\hat{\mathbf{x}} + \sin\beta(t)\hat{\mathbf{z}}$. One may take the effective magnetic field correction as orthogonal to the instantaneous effective spin, so that one can set $\mathbf{b}(t) =$

$b_1(t)\mathbf{v}_1(t) + b_2(t)\mathbf{v}_2(t)$. Using Eq. (4) together with $\overline{\overline{\Lambda}} = \Gamma\,\hat{\mathbf{y}}\hat{\mathbf{y}}$, one obtains the time-dependent coefficients $b_1(t) = 0$ and $b_2(t) = \frac{1}{2}\Gamma\sin 2\gamma(t)$. Using the definition (11) of the effective magnetic field, one obtains the corresponding corrections for the laser pulses $\delta\Omega_p(t) = -\Gamma\sin 2\gamma(t)\cos\beta(t)$ and $\delta\Omega_s(t) = \Gamma\sin 2\gamma(t)\sin\beta(t)$. Figure 1 compares the performances of the uncorrected and corrected fast STIRAP protocols. It shows the persistence of a finite overlap between the final state and the quantum states $|1\rangle, |2\rangle$ for the uncorrected protocol. This overlap is completely canceled thanks to our procedure.

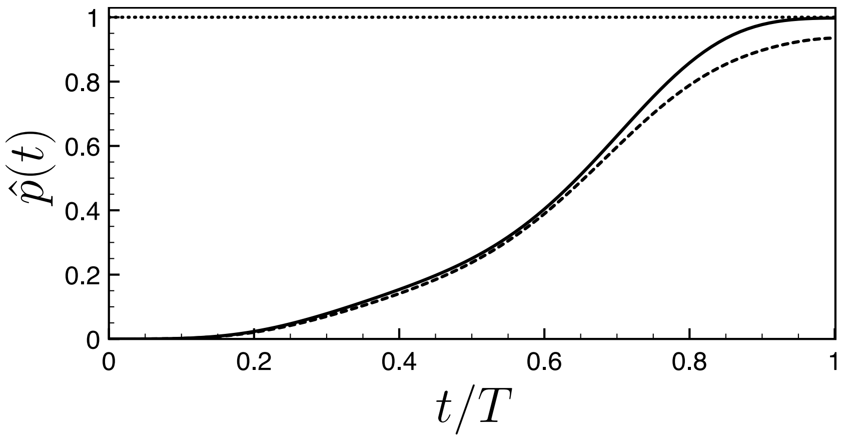

Figure 1. Fraction of the quantum state $|\psi(t)\rangle$ in the quantum state $|3\rangle$ defined as $\hat{p}(t) = |\langle 3|\psi(t)\rangle|^2/|\langle\psi(t)|\psi(t)\rangle|^2$ for the corrected (solid line) and uncorrected (dashed line) STIRAP protocols as a function of time. We have taken $\varepsilon = 0.05, \delta = \pi/4$ and ΓT=1.0 as in the main text. The horizontal dotted line helps the eye.

Preservation of the robustness to noise

We consider here the density matrix $\hat{\rho}(t)$ of a two-level atomic system with a laser field in the laser-adapted interaction picture. This interaction can be captured through the Hamiltonian

$$\hat{H}_0(t) = \frac{\hbar}{2}\begin{pmatrix} -\Delta(t) & \Omega_R(t) - i\Omega_I(t) \\ \Omega_R(t) + i\Omega_I(t) & \Delta(t) \end{pmatrix} \tag{13}$$

with a complex Rabi frequency $\Omega(t) = \Omega_R(t) + i\Omega_I(t)$ implemented by two different laser fields. As in Ref.[2], we assume the presence of independent amplitude noise components in the Rabi frequencies $\Omega_R(t), \Omega_I(t)$. This results in the stochastic Schrödinger equation

$$i\hbar\frac{d|\psi(t)\rangle}{dt} = \left[\hat{H}_0(t) + \lambda\left(\hat{H}_{2R}(t)\,\eta_1(t) + \hat{H}_{2I}(t)\,\eta_2(t)\right)\right]|\psi(t)\rangle \tag{14}$$

with delta-correlated independent stochastic functions $\eta_i(t)$ for $i = 1, 2$ such that $\langle\eta_i(t)\rangle = 0$ and $\langle\eta_i(t)\eta_j(t')\rangle = \delta_{ij}\delta(t-t')$. The Hamiltonians $\hat{H}_{2R}$ and $\hat{H}_{2I}$ correspond respectively to $\hat{H}_{2R}(t) = \frac{\hbar}{2}\Omega_R(t)\hat{\sigma}_x$ and $\hat{H}_{2I}(t) = \frac{\hbar}{2}\Omega_I(t)\hat{\sigma}_y$ with the 2×2 Pauli matrices $\vec{\hat{\sigma}}$. The averaged (in the stochastic sense) density matrix follows a master equation containing noise-induced dissipative terms, which boils down to a precession equation of the form (2) for the Bloch vector $\mathbf{S}(t) = \mathrm{Tr}\left[\hat{\rho}(t)\,\vec{\hat{\sigma}}\right]$ representing the averaged density matrix $\hat{\rho}(t)$. The effective magnetic field driving the precession is $\mathbf{B}(t) = \Omega_R(t)\hat{\mathbf{x}} + \Omega_I(t)\hat{\mathbf{y}} + \Delta(t)\hat{\mathbf{z}}$, while the dissipation tensor accounting for the laser amplitude noise yields $\overline{\overline{\Lambda}}_{\mathrm{Laser}}(t) = \frac{1}{2}\lambda^2[\Omega_I^2(t)\hat{\mathbf{x}}\hat{\mathbf{x}} + \Omega_R^2(t)\hat{\mathbf{y}}\hat{\mathbf{y}} + (\Omega_I^2(t) + \Omega_R^2(t))\hat{\mathbf{z}}\hat{\mathbf{z}}]$. Optimal shortcuts with respect to this noise have been obtained[2]. We consider an optimal shortcut respect with respect to noise optimization[2], corresponding to the Bloch vector trajectory in spherical coordinates $\theta(t) = \pi t/T - \frac{1}{12}\sin(2\pi t/T)$ and $\varphi(t) = \pi/4$. We assume the presence of an additional transverse dissipation $\overline{\overline{\Lambda}}$ given by Eq. (7) with $\Gamma_z = 0$, and consider the associated magnetic field correction (8). Finally, we perform numerical simulations of the Bloch equation

$$\frac{d\mathbf{S}}{dt} = \gamma(\mathbf{B}_0 + \mathbf{b})\times\mathbf{S} - (\overline{\overline{\Lambda}}_{\mathrm{Laser}} + \overline{\overline{\Lambda}})\,\mathbf{S} \tag{15}$$

capturing the effect of the magnetic field correction in the presence of the laser noise and of the transverse dissipation. The results are sketched on Fig. 2 of the main text for a laser noise strength corresponding to $\lambda = 1$.

Application to the fast generation of entangled states

The two-spin quantum state is driven by a spin-field interaction captured by the Hamiltonian $\hat{H}_{\mathrm{B}} = -\gamma(\hat{\mathbf{S}}_1 + \hat{\mathbf{S}}_2)\cdot\mathbf{B}(t)$, by an Ising potential $\hat{V}_{\mathrm{int}}^{(dd)} = (4\xi/\hbar)\,\hat{S}_{1z}\hat{S}_{2z}$ that accounts for the anisotropic coupling between the spins and the non-Hermitian Hamiltonian $\hat{H}_\Gamma = -i\Gamma_{|++\rangle}\,|++\rangle\langle++| - i\Gamma_{|\mathrm{Bell}\rangle}\,|\mathrm{Bell}\rangle\langle\mathrm{Bell}|$ that models the dissipation. This quantum state, which may be decomposed on the stable subspace $\{|++\rangle, |\mathrm{Bell}\rangle, |--\rangle\}$ as $|\psi(t)\rangle = a(t)|++\rangle + b(t)|\mathrm{Bell}\rangle + c(t)|--\rangle$, follows a Schrödinger equation which can be put in dimensionless form as:

$$\left\{\begin{array}{lcr} i\dot{a} & = & a(\gamma B_z + \hbar^{-1}\xi - i\Gamma_{|++\rangle}) + bB_-/\sqrt{2} \\ i\dot{b} & = & aB_+/\sqrt{2} - b(\hbar^{-1}\xi + i\Gamma_{|\mathrm{Bell}\rangle}) + cB_-/\sqrt{2} \\ i\dot{c} & = & bB_+/\sqrt{2} + c(-B_z + \hbar^{-1}\xi) \end{array}\right. \tag{16}$$

with $B_\pm = B_x \pm iB_y$. We follow the shortcut to adiabaticity procedure of Ref.[3–5]. As discussed in the main text, in order to design the shortcut and the associated correction of dissipation effects, we treat the two interacting spins as a 2D quantum system evolving in the subspace $\{|++\rangle, |\mathrm{Bell}\rangle\}$. The validity of this approach will be checked *a posteriori* by performing a numerical simulation of the Schrödinger equation on the full Hilbert space.

The shortcut is implemented with a transverse rotating field $\mathbf{B}_\perp(t) = B(t)\mathrm{Re}\left[(\hat{\mathbf{x}} + i\hat{\mathbf{y}})e^{i\omega t}\right]$ and a time-dependent longitudinal magnetic component $B_z(t)$. Switching to the interaction picture, one obtains the Hamiltonian

$$\hat{H}_{\mathrm{I}}(t) = \frac{\hbar}{2}\begin{pmatrix} \Delta(t) & \sqrt{2}\gamma B(t) \\ \sqrt{2}\gamma B(t) & -\Delta(t) \end{pmatrix} \tag{17}$$

with an effective detuning $\Delta(t) = \gamma B_z(t) - \omega + 2\xi/\hbar$. One first obtains a time-dependent Lewis-Riesenfeld invariant of the form $\hat{I}(t) = \mathbf{u}(t)\cdot\boldsymbol{\sigma}$. The time-dependent vector $\mathbf{u}(t)$ satisfies boundary conditions such that the system quantum state $|\psi(t)\rangle$ is equal at all times (up to a global phase) to the invariant eigenvector $|\phi_+(t)\rangle = \cos(\theta(t)/2)e^{i\varphi(t)}|++\rangle + \sin(\theta(t)/2)|\mathrm{Bell}\rangle$. This quantum state can be represented by a Bloch vector $\mathbf{S}_0(t)$ parametrized as in (6) by the angular functions $(\theta(t), -\varphi(t))$.

We now consider the influence of the dissipation on the evolution of the 2×2 density-matrix $\hat{\rho}(t) = |\psi(t)\rangle\langle\psi(t)|$, resulting from the Hermitian Hamitonian $\hat{H}_{\mathrm{I}}(t)$ (17) and from the anti-Hermitian Hamiltonian $\hat{H}_\Gamma(t)$:

$$i\hbar\frac{d\hat{\rho}(t)}{dt} = [\hat{\rho}(t), \hat{H}_{\mathrm{I}}(t)] + \{\hat{\rho}(t), \hat{H}_\Gamma(t)\} \tag{18}$$

where we have introduced the anticommutator $\{,\}$. The density matrix is decomposed as $\hat{\rho} = S_0\frac{\hat{\mathbb{1}}}{2} + \sum_{j=x,y,z} S_j\frac{\hat{\sigma}_j}{2}$, as well as the hermitian Hamiltonian $\hat{H}_I = \frac{\hbar}{2}\sum_{j=x,y,z}\mathscr{B}_j\hat{\sigma}_j$ and the anti-hermitian Hamiltonian $\hat{H}_\Gamma = -\frac{i\hbar}{2}(\Lambda_0\hat{\mathbb{1}} + \sum_{j=x,y,z}\Lambda_j\hat{\sigma}_j)$. The effective magnetic field $\overrightarrow{\mathscr{B}}(t)$ is expressed as a function of the control parameters as

$$\overrightarrow{\mathscr{B}}(t) = \sqrt{2}\gamma B(t)\hat{\mathbf{x}} + \Delta(t)\hat{\mathbf{z}} \tag{19}$$

and the dissipation four-vector Λ corresponds to

$$\Lambda_0 = \Gamma_{|++\rangle} + \Gamma_{|\mathrm{Bell}\rangle}, \qquad \Lambda_x = \Lambda_y = 0, \qquad \Lambda_z = \Gamma_{|++\rangle} - \Gamma_{|\mathrm{Bell}\rangle} \tag{20}$$

Using the $SU(2)$ algebra relations

$$\left[\frac{\sigma_i}{2}, \frac{\sigma_j}{2}\right] = \sum_{(i,j)\in\{x,y,z\}^2}\varepsilon_{ijk}\frac{\sigma_k}{2} \quad \text{and} \quad \{\frac{\sigma_i}{2}, \frac{\sigma_j}{2}\} = \delta_{ij}\frac{\sigma_j}{2} \tag{21}$$

(with the antisymmetric tensor ε_{ijk} such that $\varepsilon_{xyz} = 1$) into the equation of motion (18), one obtains the set of coupled differential equations:

$$\dot{S}_0 = \sum_{j=(x,y,z)}\Lambda_j S_j \tag{22}$$

$$\dot{\mathbf{S}} = \overrightarrow{\mathscr{B}} \times \mathbf{S} - \Lambda_0\mathbf{S} - S_0\overrightarrow{\Lambda} \tag{23}$$

The non-hermiticity of the Hamiltonian implies that the quantity $S_0(t) = \mathrm{Tr}[\hat{\rho}(t)]$ is no longer a constant of motion. Nevertheless, in a perturbative treatment of dissipation effects, one may take $S_0(t) = S_0(0) = 1$ to leading order. The magnetic field correction $\mathbf{b}(t)$ should fulfill a condition analogous to Eq. (4)

$$\left(\mathbf{b}\times\mathbf{S}_0(t) - \overrightarrow{\Lambda}\right)\times\mathbf{S}_0(t) = 0 \tag{24}$$

where $\mathbf{S}_0(t)$ is the dissipationless solution. Consistently with our leading order treatment of the dissipation effects, one may determine the field correction by taking $||\mathbf{S}(t)|| = 1$.

We write again the magnetic field correction $\mathbf{b}(t)$ in the spherical basis $(\mathbf{S}_0(t), \mathbf{u}_\theta(t), \mathbf{u}_\varphi(t))$ as $\mathbf{b}(t) = b_{S_0}(t)S_0(t) + b_\theta(t)\mathbf{u}_\theta(t) + b_\varphi(t)\mathbf{u}_\varphi(t)$. Note that with the considered angles $(\theta(t), -\varphi(t))$, one has $\mathbf{u}_\theta(t) = \cos\theta(t)\cos\varphi(t)\mathbf{x} - \cos\theta(t)\sin\varphi(t)\mathbf{y} - \sin\theta(t)\mathbf{z}$ and $\mathbf{u}_\varphi(t) = \sin\varphi(t)\mathbf{x} + \cos\varphi(t)\mathbf{y}$.

Condition (24) determines $b_\theta(t) = 0$ and $b_\varphi(t) = \mathbf{u}_\theta(t) \cdot \overrightarrow{\Lambda} = -\Lambda_z \sin\theta(t)$. By virtue of Eq. (19), one may only implement magnetic fields $\overrightarrow{\mathscr{B}}(t)$ such that $\overrightarrow{\mathscr{B}}(t) \cdot \hat{\mathbf{y}} = 0$. This additional constraint fixes $b_{S_0}(t) = -\Lambda_z \cos\varphi(t)/\sin\varphi(t)$, yielding the following correction for the transverse and longitudinal magnetic field components:

$$\begin{aligned} \gamma\delta B(t) &= -\frac{\Gamma_{|++\rangle} - \Gamma_{|\mathrm{Bell}\rangle}}{\sqrt{2}} \sin\theta(t)\sin\varphi(t)\left(1 + \frac{1}{\tan^2\varphi(t)}\right) \\ \gamma\delta B_z(t) &= (\Gamma_{|++\rangle} - \Gamma_{|\mathrm{Bell}\rangle})\frac{\cos\theta(t)}{\tan\varphi(t)} + \omega - \frac{2\xi}{\hbar} \end{aligned} \tag{25}$$

For the numerical simulations of the full Schrödinger equation, we have considered the following shortcut involving the time-dependent magnetic field[4,5]

$$\gamma B(t) = \frac{\dot{\theta}(t)}{\sqrt{2}\sin\varphi(t)} \qquad \gamma B_z(t) = -\dot{\varphi}_0(t) + \frac{\dot{\theta}_0(t)}{\tan\theta(t)\tan\varphi(t)} + \omega - \frac{2\xi}{\hbar} \tag{26}$$

with angular functions satisfying adequate boundary conditions in order to avoid divergent fields

$$\theta(t) = -3\pi\left(\frac{t}{T}\right)^2 + 2\pi\left(\frac{t}{T}\right)^3 \qquad \varphi(t) = -\pi/2 - \pi\left(\frac{t}{T}\right) + 5\pi\left(\frac{t}{T}\right)^2 - 8\pi\left(\frac{t}{T}\right)^3 + 4\pi\left(\frac{t}{T}\right)^4 \tag{27}$$

We have taken $\omega T = 2$. The magnetic field correction is obtained directly from Eq. (25).

References

1. Chen, X. & Muga, J. G. Engineering of fast population transfer in three-level systems. *Phys. Rev. A* **86**, 033405 (2012).
2. Ruschhaupt, A., Chen, X., Alonso, D. & Muga, J. G. Optimally robust shortcuts to population inversion in two-level quantum systems. *New J. Phys.* **14**, 093040 (2012).
3. Unanyan, R. G., Vitanov, N. V. & Bergmann, K. Preparation of entangled states by adiabatic passage. *Phys. Rev. Lett.* **87**, 137902 (2001).
4. Paul, K. & Sarma, A. K. High-fidelity entangled bell states via shortcuts to adiabaticity. *Phys. Rev. A* **94**, 052303 (2016).
5. Zhang, Q., Chen, X. & Guéry-Odelin, D. Reverse engineering protocols for controlling spin dynamics. *Sci. Reports* **7**, 15814 (2017).
